# COVID-19 Vaccine–Related Discussion on Twitter: Topic Modeling and Sentiment Analysis

**DOI:** 10.2196/24435

**Published:** 2021-06-29

**Authors:** Joanne Chen Lyu, Eileen Le Han, Garving K Luli

**Affiliations:** 1 Center for Tobacco Control Research and Education University of California, San Francisco San Francisco, CA United States; 2 School of Information University of Michigan Ann Arbor, MI United States; 3 Department of Mathematics University of California, Davis Davis, CA United States

**Keywords:** COVID-19, vaccine, vaccination, Twitter, infodemiology, infoveillance, topic, sentiment, opinion, discussion, communication, social media, perception, concern, emotion

## Abstract

**Background:**

Vaccination is a cornerstone of the prevention of communicable infectious diseases; however, vaccines have traditionally met with public fear and hesitancy, and COVID-19 vaccines are no exception. Social media use has been demonstrated to play a role in the low acceptance of vaccines.

**Objective:**

The aim of this study is to identify the topics and sentiments in the public COVID-19 vaccine–related discussion on social media and discern the salient changes in topics and sentiments over time to better understand the public perceptions, concerns, and emotions that may influence the achievement of herd immunity goals.

**Methods:**

Tweets were downloaded from a large-scale COVID-19 Twitter chatter data set from March 11, 2020, the day the World Health Organization declared COVID-19 a pandemic, to January 31, 2021. We used R software to clean the tweets and retain tweets that contained the keywords *vaccination*, *vaccinations*, *vaccine*, *vaccines*, *immunization*, *vaccinate*, and *vaccinated*. The final data set included in the analysis consisted of 1,499,421 unique tweets from 583,499 different users. We used R to perform latent Dirichlet allocation for topic modeling as well as sentiment and emotion analysis using the National Research Council of Canada Emotion Lexicon.

**Results:**

Topic modeling of tweets related to COVID-19 vaccines yielded 16 topics, which were grouped into 5 overarching themes. Opinions about vaccination (227,840/1,499,421 tweets, 15.2%) was the most tweeted topic and remained a highly discussed topic during the majority of the period of our examination. Vaccine progress around the world became the most discussed topic around August 11, 2020, when Russia approved the world’s first COVID-19 vaccine. With the advancement of vaccine administration, the topic of instruction on getting vaccines gradually became more salient and became the most discussed topic after the first week of January 2021. Weekly mean sentiment scores showed that despite fluctuations, the sentiment was increasingly positive in general. Emotion analysis further showed that trust was the most predominant emotion, followed by anticipation, fear, sadness, etc. The trust emotion reached its peak on November 9, 2020, when Pfizer announced that its vaccine is 90% effective.

**Conclusions:**

Public COVID-19 vaccine–related discussion on Twitter was largely driven by major events about COVID-19 vaccines and mirrored the active news topics in mainstream media. The discussion also demonstrated a global perspective. The increasingly positive sentiment around COVID-19 vaccines and the dominant emotion of trust shown in the social media discussion may imply higher acceptance of COVID-19 vaccines compared with previous vaccines.

## Introduction

As the COVID-19 pandemic spread globally, COVID-19 vaccine-related issues received increasing public attention. Multiple research teams in major pharmaceutical companies and research institutions across various nations have been developing vaccines [1,2]. Although vaccination is a cornerstone of the prevention of communicable infectious diseases [3], the practice has traditionally faced public fears, hesitancy, and even opposition [4,5]. During the COVID-19 pandemic, it is estimated that 55% to 85% of the population, depending on the country and the infection rate, needs to receive the COVID-19 vaccine to provide herd immunity [6,7]. However, a survey about COVID-19 vaccine intentions in September 2020 suggested that 21% of the public in the United States would definitely get vaccinated and 24% would definitely not get vaccinated [8]. One factor leading to the low acceptance of vaccines is poor health literacy, which is significantly influenced by social media use [9]. Therefore, there is a pressing need to understand how COVID-19 vaccines have been discussed on social media to better understand the public perceptions, concerns, and sentiments that may influence the achievement of herd immunity goals.

Although social media data analysis has been widely performed for both health-related issues and emerging public health crises [10-14], analysis of big data of social media discussion on COVID-19 vaccines is limited [15,16]. To the best of our knowledge, in the most recently published work about COVID-19 vaccine–related social media discussion, the study period ended in November 2020 [16]. However, many significant events related to COVID-19 vaccines have occurred since that date, such as the confirmation of more COVID-19 variant cases in North America by the US Centers for Disease Control and Prevention (CDC), rollout of vaccines, and increased numbers of vaccines showing high efficacy. Previous studies found that changes in social media discussion about vaccine-related topics correspond to the changing reality [15,17]. Thus, research involving recent social media data is needed to fully understand the public discussion on COVID-19 vaccines during the pandemic. In addition, knowledge of the content of COVID-19 vaccine discussion on social media will provide a possible explanation for users’ attitudes toward COVID-19 vaccines and COVID-19 vaccine acceptance or hesitancy. However, previous research on COVID-19 vaccines did not touch on these topics [16,18-21]. To fill this gap, in this study, we will examine the public discourse about COVID-19 vaccines on Twitter since the World Health Organization announced it to be a global pandemic on March 11, 2020, up to January 31, 2021, to identify the topics, overarching themes, and sentiments around COVID-19 vaccines and vaccination. This is the first study to include data from almost one year of the pandemic. This long time span will allow us not only to observe a bigger picture of the public discussion and concerns regarding COVID-19 vaccines but also discern the salient changes in major topics and sentiments during the course of the pandemic and further inform public health education and campaigns for increasing COVID-19 vaccine acceptance. Also, our results may provide insight that will be useful in the promotion of other vaccines.

## Methods

### Data Extraction and Preprocessing

The IDs from a total of 1,499,421 tweets, without retweets, from March 11, 2020, through January 31, 2021, were obtained using the data set maintained by Georgia State University’s Panacea Lab [22]. These tweets were collected by the Panacea Lab using the following 13 keywords: *COVID19*, *CoronavirusPandemic*, *COVID-19*, *2019nCoV*, *CoronaOutbreak*, *coronavirus*, *WuhanVirus*, *covid19*, *coronaviruspandemic*, *covid-19*, *2019ncov*, *coronaoutbreak*, and *wuhanvirus*. The Panacea Lab can only provide the tweet IDs [23], which need to be hydrated to recover the full tweet data.

During the tokenizing stage, we used the *gsub* function in R (R Foundation for Statistical Computing) to extract the tweets whose language field in the tweets’ metadata was specified as English. All text mining was performed using RStudio Version 1.4.1103 on a Mac computer (Apple Inc) running Big Sur, version 11.2.2. We converted the text of all the tweets to lowercase. We further filtered the tweets by the following keywords: *vaccination*, *vaccinations*, *vaccine*, *vaccines*, *immunization*, *vaccinate*, and *vaccinated*. We prepared 2 batches of tweets, 1 for text mining and 1 for sentiment/emotion analysis. The data processing procedures for the 2 batches are almost the same except at the beginning: for sentiment analysis, we converted all the emojis to words, whereas for text mining, we removed all the emojis. Next, we created a script to remove the URLs, mentioned names, non–American Standard Code for Information Interchange (ASCII) characters, and anything other than English letters or spaces (eg, “1” and “?”). Using the R package *dplyr*, version 1.0.2, we cleaned the tweets by removing duplicates. To filter tweets created by fake or bot accounts, we used the document-term matrix (DTM), which contains rows corresponding to the tweets and columns corresponding to the terms. Each entry in the DTM denotes the number of times a term appears in a tweet. The similarity matrix *S*=[*S_ij_*], which measures how similar the *i*-th tweet is to the *j*-th tweet, is obtained by computing the dot product between the *i*-th row vector and the *j*-th row vector *R_j_* in the DTM, which geometrically represents the cosine of the angle between the row vectors *R_i_*, *R_j_*:



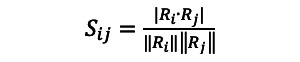



Therefore, if the *i*-th and the *j*-th tweets are identical, then *S_ij_*=1; if they are completely different (ie, the angle between the corresponding row vectors is 90º), then *S_ij_*=0. For tweets that were 80% similar, we retained the most representative one (measured by the magnitude of the row vector in the DTM). Furthermore, we used the *tweetornot* package [24], version 0.1.0, to remove users that were identified as bots with a 95% probability or higher.

**Figure 1:**
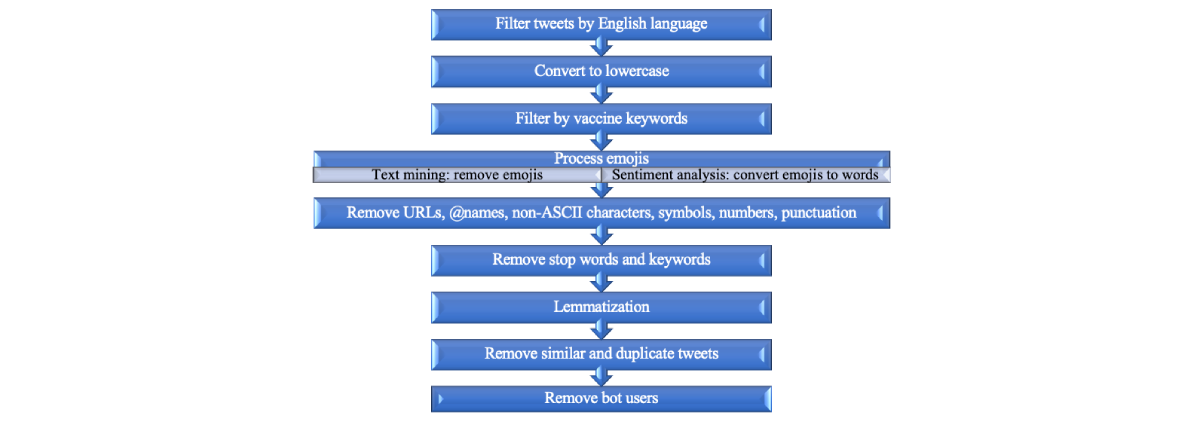
Data preprocessing procedure. ASCII: American Standard Code for Information Interchange.

The final cleaned data set consisted of 1,499,421 unique tweets from 583,499 different users. We further cleaned the tweets by removing words and characters that were of little or no analytical value (eg, “the”, “very”, “&”). We performed this task by creating our own list of stop words by appending the 13 keywords related to *COVID-19* and the 7 keywords related to *vaccine* to the English stop words list from the R package *tidytext*, version 0.2.6; this step was performed because we already knew that every tweet would contain one or more of those keywords, and retaining them in the tweets would not further our understanding of the main content of the tweets. Finally, we stemmed and lemmatized the words to their root forms using the R package *textstem*, version 0.1.4 (eg, *vaccinating*, *vaccinates*, and *vaccinated* were changed to *vaccinate*). Figure 1 shows a summary of our data preprocessing procedure.

### Topic Modeling

Topic modeling can help organize a large collection of documents by grouping documents into different themes. Topic modeling is often referred to as a probabilistic clustering. It is more robust and usually provides more realistic results than hard clustering (eg, k-mean clustering) [25]. A typical clustering algorithm assumes a distance measure between topics and assigns one topic to each document, whereas topic modeling assigns a document to a collection of topics with different weights or probabilities without any assumption of the distance measured between topics. Many topic models are available, of which the most widely used is the latent Dirichlet allocation (LDA) model [25], developed by David Blei, Andrew Ng, and Michael I Jordan in 2002 [26].

To extract common topics from this very high number of tweets, we used the LDA algorithm for topic modeling. We performed the LDA algorithm on the data using the R *textmineR* package, version 3.0.4. The LDA algorithm requires manual input of the number of expected topics. We ran the LDA algorithm on the data by varying the topic number from 2 through 40. For each topic number, we calculated the coherence score using the *textmineR* package. We chose 16 as the topic number for our final topic model, based on two considerations: first, the topic number 16 corresponded to the highest coherence score (see Multimedia Appendix 1); second, in comparison with topics that appeared in the other topic models, the topic model with 16 topics strikes a balance between one too narrow that the model would risk excluding important topics and one so broad that it would dilute the main focus.

**Figure 2:**
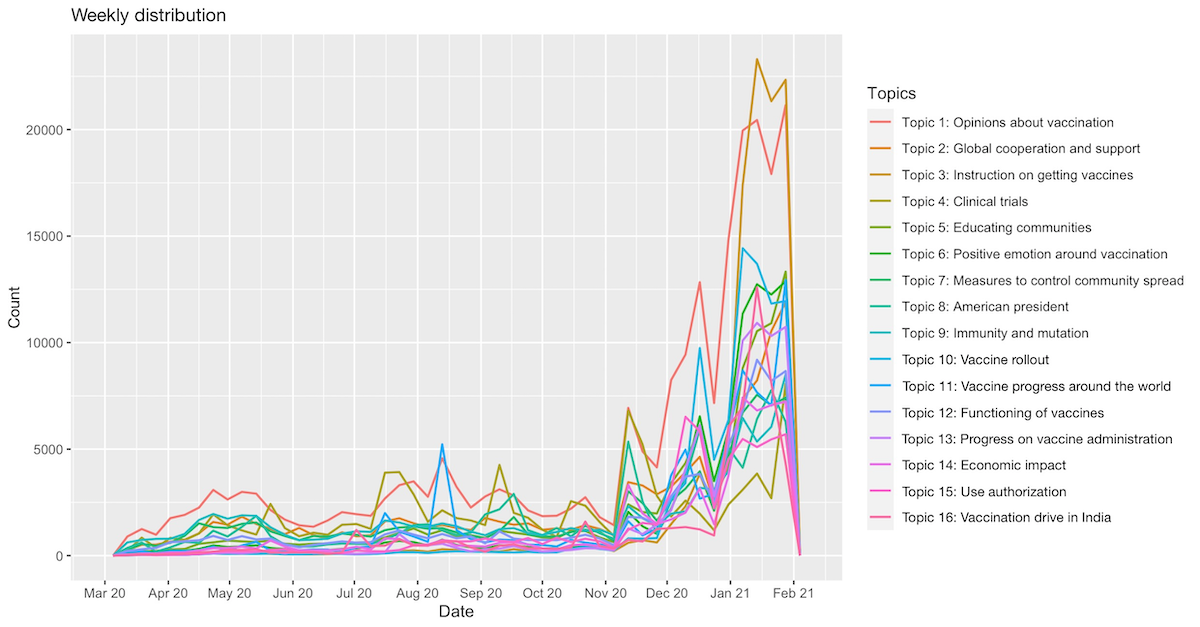
Weekly frequency of each topic on Twitter from March 11, 2020, to January 31, 2021.

The top 8 terms from each of the 16 topics were generated. We also used the *geo_freqpoly* function in the R package *ggplot2*, version 3.3.2, to generate the frequency polygons (see Figure 2) to visualize the weekly frequency of the 16 topics from March 11, 2020, to January 31, 2021. For each tweet, the LDA assigned a probability to each of the 16 topics. We assigned the topic with the highest probability to each tweet, and we grouped the tweets according to the most prevalent topics. To obtain representative tweets for each topic, we randomly sampled 100 tweets from each topic; two of the authors then independently examined the sampled tweets, followed by a group discussion to select the most representative ones. If one of the authors thought that no conspicuous topics had emerged from the first 100 sampled tweets, another 100 tweets would be sampled and further reviewed; the authors continued this process until they judged that there was a clear common topic and they reached a consensus (see our previous paper [27] for more details). We used the *textmineR* package’s topic label function to generate an initial labeling for the topics. After carefully reading through the sampled tweets from each topic, the two authors refined the machine-generated labeling to give each topic the most accurate, concise, and coherent description (see Table 1 in the *Results* section). Through discussions, the authors further grouped the topics into 5 overarching themes. Specifically, two of the authors first independently grouped the topics into the number of themes that made the most sense to them and resolved conflicting views through discussion. The third author was involved by providing additional comments on both the agreements and disagreements between the two authors. The final decision of the grouping was made together by all three authors. For example, whether the topic of “vaccination drive in India” should be grouped into the theme of “vaccine administration” or “vaccines as a global issue” was unresolved after the two-author discussion. By rereading tweets and discussing among the three authors, we finally placed the topic under the theme of vaccines as a global issue.

### Sentiment and Emotion Analyses

**Figure 3:**
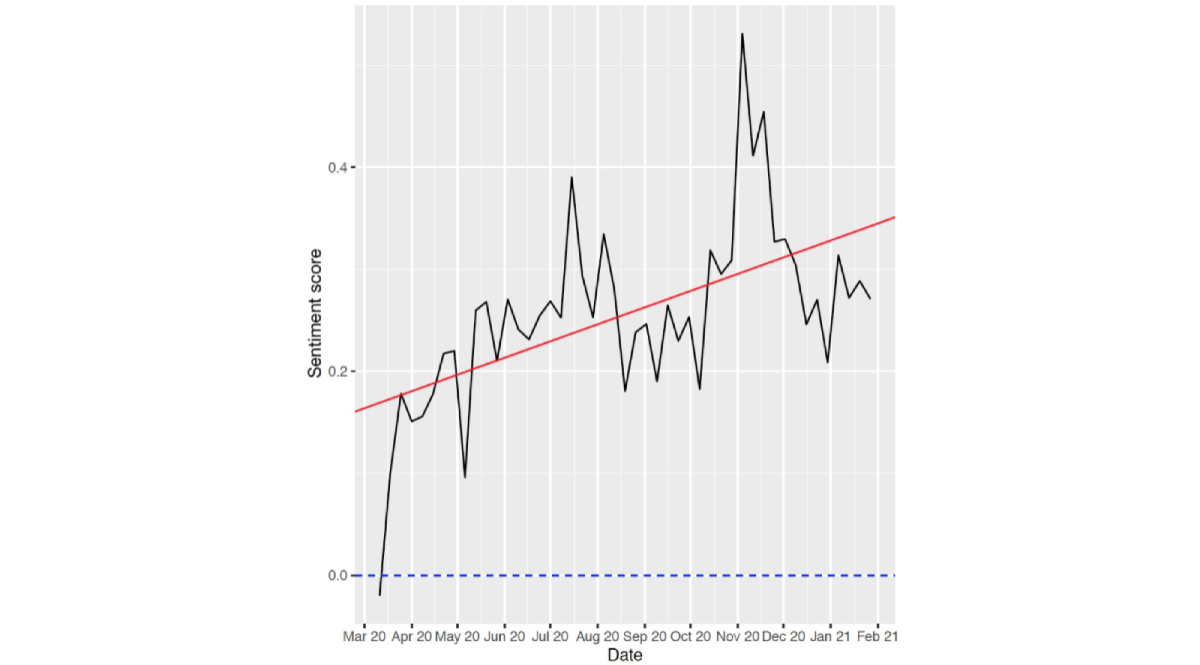
Weekly average polarity (sentiment) scores from March 11, 2020, to January 31, 2021. The slope of the best fit is 0.003764.

**Figure 4:**
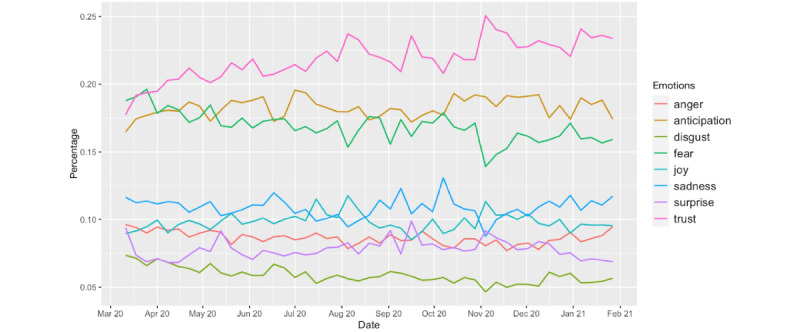
Weekly percentages of emotions from March 11, 2020, to January 31, 2021.

Sentiment analysis can be used to classify the polarity of a given document; it can assign a score to a document to indicate whether the expressed opinion is positive, negative, or neutral. Emotion analysis goes beyond simple polarity and can give scores to different emotions, such as anger, fear, anticipation, trust, surprise, sadness, joy, and disgust (the so-called Plutchik wheel of emotions) [28]. The *syuzhet* package (Jockers, 2017) [29] is the most popular and efficient R package for sentiment/emotion analysis. To perform the emotion analysis, we used the National Research Council of Canada Emotion Lexicon developed by Turney and Mohammad in 2010 [30]. It is the most comprehensive dictionary for this task [31]. In Figure 3, we show the weekly average polarity (sentiment) scores from March 11, 2020, to January 31, 2021; we fitted the data points with the best linear fit and obtained a slope of 0.003764 with a *P* value <.001 and an intercept of 0.1653927 with a *P* value <.001. In Figure 4, we plot the weekly percentage of emotions.

## Results

### Numbers of Tweets

**Figure 5:**
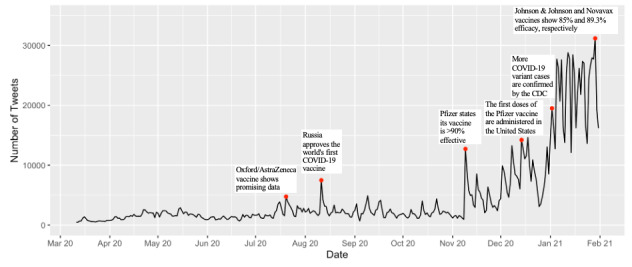
Daily numbers of COVID-19–related tweets from March 11, 2020, to January 31, 2021.

We downloaded 144,332,894 tweets posted from March 11, 2020, through January 31, 2021 (for a total of 327 days) using the Panacea Lab database. After cleaning, a total of 1,499,421 tweets from 583,499 different users were included in the analysis. As shown in Figure 5, the number of daily tweets continually increased; the daily average for the month of January 2021 was 22,202 tweets. Before November 9, 2020, the number of daily tweets was around or below 5000, with only one exception, on August 11, 2020 (n=7486), when Russia approved the world’s first COVID-19 vaccine [32]. The first wave of an exponential increase in the number of daily tweets started on November 9, 2020 (n=12,720), when Pfizer stated that its vaccine is 90% effective [33]. The second wave of a surge in the number of daily tweets started around January 3, 2021, when more COVID-19 variant cases were confirmed in North America by the CDC, and the highest number of tweets in a single day (n=31,197) occurred on January 29, 2021, when Johnson & Johnson’s and Novavax’s vaccines showed 85% and 89.3% efficacy, respectively [34].

### Topic Modeling of COVID-19 Vaccine–Related Tweets

Analysis of the tweets yielded 16 topics, which were grouped into 5 overarching themes. In descending order of quantity of tweets (N=1,499,421), the themes are opinions and emotions around vaccines and vaccination (405,397 tweets, 27.04%), knowledge around vaccines and vaccination (355,305 tweets, 23.7%), vaccines as a global issue (311,251 tweets, 20.76%), vaccine administration (266,744 tweets, 17.79%), and progress on vaccine development and authorization (160,724 tweets, 10.72%). Table 1 summarizes the 16 topics, top terms in the topics, and the number and percentage of each topic; it also provides a tweet example for each topic. More details about the themes and topics, including the salient temporal variance of topics (see Figure 2), are elaborated in the following subsections.

**Table 1:** Numbers and percentages of tweets related to each COVID-19 vaccine topic (N=1,499,421).

Theme and topics		Top terms contributing to the topic model	Total tweets, n (%)	Example paraphrased tweet^a^ (date posted)
**Theme 1: Opinions and emotions around vaccines and vaccination**				
	Opinions about vaccination	*people*, *get*, *take*, *go*, *want*, *make*, *like*, *think*	227,840 (15.20)	“It is pathetic to see the low trust in science and the government. People need to see leaders and politicians on TV to receive the vaccine to convince them that the vaccine is safe.” (December 18, 2020)
	Positive emotion around vaccination	*get*, *good*, *first*, *day*, *today*, *work*, *one*, *feel*	89,117 (5.94)	“After getting the first dose of the covid-19 vaccine today, I can finally breath some fresh air and feel there is hope in life.” (January 23, 2021)
	American president	*Trump*, *Biden*, *American*, *president*, *gate*, *bill*, *plan*, *administration*	88,440 (5.90)	“There are more lies than usual in today’s press. Biden is being portraited as anti-vaccine. This is not correct. In fact, he said on Wednesday he trusted the vaccines and the scientists, while accusing President Trump of playing politics with a potential covid-19 vaccine.” (September 16, 2020)
**Theme 2: Knowledge about vaccines**				
	Educating communities	*question*, *read*, *answer*, *expert*, *article*, *community*, *immunity*, *black*	96,532 (6.44)	“You can learn more about the covid-19 vaccines in our upcoming town hall meeting. We will address any questions and concerns on January 13, 2021, 2-3:30 PM (EST).” (January 7, 2021)
	Measures to control community spread	*mask*, *get*, *need*, *go*, *still*, *available*, *end*, *life*	89,008 (5.94)	“Even though covid-19 vaccines are an important step in slowing down the spread of the virus, people still need to continue taking all precautions: wear a mask, maintain physical distance from others, and keep your hands clean.” (January 19, 2021)
	Immunity and mutation	*flu*, *year*, *new*, *may*, *variant*, *work*, *effective*, *strain*	88,327 (5.89)	“They say that coronavirus resembles the flu virus. As we know, the flu virus mutates. Therefore, they need to create different flu shot every year into order to fight off the virus, but there hasn’t been a flu vaccine that is 100% effective. So good luck with making the covid-19 vaccine.” (May 11, 2020)
	Functioning of vaccines	*people*, *effect*, *test*, *risk*, *side*, *side effect*, *immune*, *death*	81,438 (5.43)	“Both vaccines use mRNA technology which contains instructions that tell our cells how to make a protein that triggers an immune response inside our bodies.” (December 21, 2020)
**Theme 3: Vaccines as a global issue**				
	Global cooperation and support	*world*, *country*, *global*, *pandemic*, *access*, *need*, *develop*, *effort*	108,366 (7.23)	“On Thursday, WHO [the World Health Organization] warns against ‘vaccine nationalism.’ No one country is safe if poor countries can’t get access to the vaccines.” (August, 9, 2020)
	Vaccine progress around the world	*UK*, *China*, *EU*, *Russia*, *first*, *country*, *approve*, *news*	83,156 (5.55)	“On Tuesday, German health minister Jens Spahn joined EU [the European Union] to place restriction on exporting covid-19 vaccines. This came amid discontent over the delay in rolling out the AstraZeneca vaccine to member countries.” (January 26, 2021)
	Economic impact	*new*, *case*, *death*, *news*, *rollout*, *rate*, *high*, *report*	61,360 (4.09)	“A rise in new covid-19 cases outweigh positive vaccine news. Shutdown fears sink global markets. US coronavirus deaths are above 250,000.” (November 9, 2020)
	Vaccination drive in India	*India*, *drive*, *minister*, *Indian*, *speed*, *warp*, *health*, *warp speed*	58,369 (3.89)	“India clears covid-19 vaccine makers Serum Institute of India (SII) and Bharat Biotech to start the world’s largest immunization drive.” (January 3, 2021)
**Theme 4: Vaccine administration**				
	Instruction on getting vaccines	*county*, *health*, *state*, *appointment*, *update*, *plan*, *site*, *distribution*	106,544 (7.11)	“Health departments will each announce their vaccine availability and locations. Eligible individuals at the federally qualified health center will be contacted regarding access to a covid-19 vaccine.” (December 30, 2020)
	Vaccine rollout	*worker*, *care*, *health*, *first*, *receive*, *healthcare*, *hospital*, *staff*	85,623 (5.71)	“We have many sites delivering the covid-19 vaccines to the top priority group. Please be patient and if you are in the top priority group, you will be contacted.” (January 17, 2021)
	Progress on vaccine administration	*dose*, *million*, *first*, *receive*, *week*, *Pfizer*, *people*, *first dose*	74,577 (4.97)	“Since Tuesday’s update, Louisiana has administered 25,133 additional covid-19 vaccines. The second doses started being administered this week. 7,068 people have been fully vaccinated. In total, since mid-December, 81,585 doses have been administered.” (January 7, 2021)
**Theme 5: Progress on vaccine development and authorization**				
	Clinical trials	*trial*, *clinical*, *Pfizer*, *Moderna*, *phase*, *effective*, *clinical trial*, *oxford*	99,754 (6.65)	“There are 70 covid-vaccines under development. Moderna is one of the first to test their covid-19 vaccine on humans. If the trial is successful, Moderna could reach the final stage of testing by Fall 2020.“ (April 26, 2020)
	Use authorization	*use*, *FDA*, *approve*, *emergency*, *approval*, *Pfizer*, *emergencyuse*, *health*	60,970 (4.07)	“The FDA’s [US Food and Drug Administration’s] vaccine advisory committee has unanimously approved an emergency use of the covid-19 vaccine made by Pfizer and by Moderna.” (January 19, 2021)

^a^The tweets are paraphrased to protect users’ privacy.

### Theme 1: Opinions and Emotions Around Vaccines and Vaccination

This theme contained 3 topics, among which the topic of opinions about vaccination (227,840/1,499,421 tweets, 15.2%) was the most tweeted of all the 16 topics and remained the most discussed topic in the majority of the period of our examination (see Figure 2). This theme reflected the mixed opinions of Twitter users about vaccination, including their doubt, hesitancy, trust, and advocacy. Some Twitter users also asked for freedom about vaccination. The second topic, positive emotion around vaccination, featured happiness, hope, relief and other positive emotions that the public showed in their tweets. Many emotions were based on their direct or indirect experiences with vaccination. The third topic was named “American president.” The public’s opinions and emotions were expressed through their comments on US President Trump and President Biden’s vaccine-related words and actions.

### Theme 2: Knowledge Around Vaccines and Vaccination

This theme consisted of 4 topics focusing on understanding and facilitating understanding of COVID-19 vaccines and vaccination. The most frequently discussed topic under this theme was educating communities, with a considerable number of tweets spreading information about the live webinars where health professionals would provide important information about COVID-19 vaccines and answer questions. The topic of measures to control community spread featured the recommendation of taking measures such as wearing a mask, washing hands, and social distancing both before and after getting vaccinated. The topics of immunity and mutation centered around the immunity of vaccines and mutation of the coronavirus, which was frequently discussed in comparison with *flu virus*. Functioning of vaccines, as a topic, introduced how vaccines work and the subsequent symptoms and side effects after vaccination.

### Theme 3: Vaccines as a Global Issue

The theme of vaccines as a global issue consisted of 4 topics related to the globality of SARS-CoV-2 and the COVID-19 vaccines; therefore, the topics under this theme were not centered on the United States. These topics involved active news about vaccine progress and updates around the world, such as international vaccine supplies, delivery, and purchases in many countries (ie, the topic of vaccine progress around the world), the impact of vaccines on the global economy (ie, the topic of economic impact), and the world’s largest inoculation drive (ie, the topic of the vaccination drive in India). The topic of vaccine progress around the world became the most discussed topic around August 11, 2020, because Russia approved the world’s first COVID-19 vaccine (see Figure 2). The most salient topic under this theme was global cooperation and support, which called for global cooperation to accelerate the vaccine development and equitable access and advocated no vaccination nationalism. This was also the second most tweeted topic (108,366/1,499,421 tweets, 7.23%) among the 16 topics, only after the topic of opinions about vaccination.

### Theme 4: Vaccine Administration

This theme consisted of 3 topics covering several aspects of vaccine administration. The topic of vaccine rollout mainly centered around vaccination to the top priority groups, including health care providers being the first in line. After the first doses of Pfizer vaccine were administered in the United States on December 14, this topic remained one of the 3 most discussed topics for approximately 5 weeks (see Figure 2). The second topic focused on the progress of the vaccine administration, including the shipment and supply of vaccines that directly relate to the progression of vaccine administration. The third topic under this theme was instruction on obtaining vaccines, which included spreading of information from health authorities at various levels to guide the public to obtain their vaccine shots. More detailed information was mentioned in the tweets in this topic, such as “If anyone is 75 and older, does not have internet access, and needs help to schedule an appointment, please call the central appointment desk for help at 1-866-960-0633” (a tweet posted on January 20, 2021). With the advancement of vaccine administration, this topic gradually became more salient and leaped to the rank of most discussed topic, surpassing the topic of opinions about vaccination, after the first week of January; it then remained at the top rank until the end of January (see Figure 2).

### Theme 5: Progress on Vaccine Development and Authorization

There are 2 topics under this theme, which focused on the development of COVID-19 vaccines and authorization by the US Food and Drug Administration (FDA). The topic of clinical trials centered around the plan and process of clinical trials, mainly from Pfizer and Moderna, and the updated results of the clinical trials. This topic remained the most discussed topic for a week around July 20, when the first human trial of the Oxford/AstraZeneca coronavirus vaccine showed promise [35], and became 1 of the 2 most discussed topics, together with the topic of opinions about vaccination, for 3 weeks after Pfizer stated its vaccine is 90% effective on November 9, 2020 (see Figure 2). The topic of use authorization centered on FDA approval of an emergency use authorization for COVID-19 vaccines.

### Sentiment and Emotion Analyses of COVID-19 Vaccine Tweets

Weekly mean sentiment scores showed that despite fluctuations, in general, the sentiment was increasingly positive from March 11, 2020, to January 31, 2021, with the linear best fit slope of 0.003764 (with a *P* value <.001, which indicates that it is statistically significant) (see Figure 3). Moreover, the positive emotion reached its peak around November 9, 2020, when Pfizer announced that its vaccine is 90% effective; on the same day, the number of daily tweets became historically high before January, as mentioned above. Emotion analysis further showed that trust was the most predominant emotion, accounting for 22.78% of the 8 emotions (anger, anticipation, disgust, fear, joy, sadness, surprise, and trust), followed by anticipation (18.34%), fear (16.29%), sadness (10.97%), joy (9.76%), anger (8.63%), surprise (7.60%), and disgust (5.63%). Noticeably, the most dominant emotion shown in COVID-19 vaccine tweets before April was fear; however, it changed to trust from the week of April 1, 2020 (Figure 4). Trust remained the most dominant emotion after that, and the number of tweets expressing trust continued to grow. In addition, it was observed that when the emotion of trust increased, the emotion of fear decreased. The trust emotion reached its peak on November 9, 2020, when Pfizer announced that its vaccine is 90% effective; on the same day, the fear emotion was expressed the least throughout the time period of our examination. It was also noted that apart from the obvious changes in the emotions of trust and fear over time, the other emotions were relatively stable in the period of March 11, 2020, to January 31, 2021 (see Figure 4).

## Discussion

### Principal Findings

In this study, we examined sentiments and topics over a long time span, covering the discussions about COVID-19 vaccines from when COVID-19 became a global pandemic (March 11, 2020) to January 31, 2021, when multiple vaccines become available and mass vaccination had begun in the United States and many other countries. This study adds to the latest research about the social impact of the COVID-19 vaccine. For example, there are surveys addressing sociodemographic social media user characteristics and the social determinants of vaccine acceptance [18,36]. Our research supplements studies like these by providing the discourse patterns on social media, that is, how people actually talked about their vaccination intentions and other related issues. Researchers have taken a similar approach to studying very specific vaccine-related topics, such as side effects and the type of vaccines in the context of China. This study could provide a comparison in different cultural contexts, as the corpus was pulled from a global data set and English tweets were analyzed. The specific topics and public sentiments identified could be used for further studies about specific vaccine-related topics among English language social media users. Many of the current studies about COVID-19 vaccination focus on vaccine hesitancy and antivaccine messages [19], and some used survey methods to gather data [37]. As the vaccines for COVID-19 are still very new, and they were developed in a very short period of time due to the urgent need, we would expect the expression of vaccine hesitancy in the public media channels. This study would help contextualize the major concerns about vaccine efficacy and safety.

This study has found some changing patterns of the discussion on Twitter along with the progress. Similar patterns of sentiments and topics found in another research using natural language processing and deep learning techniques on Facebook and Twitter posts [16]. The results of this research show that the patterns are valid across platforms. The number of tweets regarding COVID-19 vaccination is largely driven by major events—mainly the milestones in vaccine development and the new variants of the virus. Major spikes in the number of tweets correspond closely to these events. The analysis of each topic also shows such patterns. For example, from the weekly distribution of topics, a sudden overall increase is noticeable starting from early November, also right at the time of the Pfizer announcement. Scholars have studied how information flows from social media to mainstream news. In today’s media ecology, the boundary between social and mainstream media is no longer clear; however, here we can see that social media discourse is largely mirroring what is happening in the news. Future studies could detect to what extent the social media discussion is shaped by mainstream media.

The sentiment analysis shows that the general sentiment toward COVID-19 vaccination is becoming more positive over time. The overall sentiment score reached the maximum in early November 2020, which also corresponded to the report of the high efficacy of the Pfizer vaccine. As for the emotions, trust continually dominated the discussion; it reached the highest point around early November, following Pfizer’s announcement about the efficacy of its vaccine. The percentage of tweets increased overall, showing that more people were expressing their trust in the discussion of COVID-19 vaccine. The change of the percentage of tweets expressing fear mirrors that of tweets expressing trust. The overall percentage is decreasing, showing that as the vaccine development progressed, people’s fear about the pandemic decreased. The highest point was in mid-March 2020, after the declaration of the global pandemic, and the lowest point was in early November, at the time of Pfizer’s announcement. As vaccine research and testing moved closer to a promising result, the expression of fear declined. Other emotions, in terms of their percentages of the overall tweets, remained more or less stable over time. Trust is the dominant emotion, which can be understood as a reflection of the vaccination as the only option. Unlike other types of vaccines, which people can choose to take or not, vaccination has been increasingly viewed as the only promising way to end the pandemic given the prevalence of COVID-19, the speed of its community spread, the disruption of normal life, and the lack of other options proved to be efficient.

The topics related to opinions and emotions were the most common, and among the 3 topics in this theme, the topic on opinions about vaccination represented the largest proportion. The development of COVID-19 vaccines has progressed along with the spread of the virus and the appearance of variants, together with our increasing knowledge about the disease, all of which have become active topics in public discourse. With many uncertainties remaining about vaccines and other options, we would expect mixed opinions to surface on the platform. This mixed opinion about vaccination should also be situated in the larger context of the antivaccine movement in the United States and other countries as well. Our results show that doubts, vaccine hesitancy, conspiracy theories, and the argument that vaccination is an individual freedom are all common themes in the antivaccine discourse. However, as mentioned earlier, as COVID-19 has affected people’s day-to-day life, a vaccine is crucial for returning to normal life; people still have much hope in vaccines, leading to the sharing of positive emotions. Finally, with the political climate and the ongoing presidential election, both candidates included vaccination on their agenda; thus, the discussion about the vaccine was very politicized.

As COVID-19 is an ongoing crisis with a global scale, the discussion about the vaccine is also global. The pandemic has revealed how much the world is now connected, and thus vaccination has become a global issue—if a country cannot reach a certain level of vaccination of its population, it has a high risk of contagion and virus mutation; thus, it is difficult for the country to return to its role in the global economy, and global cooperation is needed to defeat the disease. Therefore, the economic impact of the pandemic and the development of vaccines are salient issues.

The currently available vaccines are also results of cross-national collaboration, which is why “vaccine nationalism” is frequently mentioned in tweets as an issue that would impede the progress of fighting the pandemic. In a global crisis, information-seeking needs increase, particularly as vaccines become available and people need instructive information regarding the vaccine rollout, obtaining vaccines, and the vaccine administration. As COVID-19 is a new disease that is still being studied, and the vaccines also became available after a short period of research and development, there are numerous uncertainties facing public acceptance of these vaccines. For laypeople, the science behind the vaccines is still not well understood, particularly because COVID-19 is such a new disease and many things are still unknown. These uncertainties have provoked people to seek and share information about the vaccines, which is reflected in the topic of knowledge around vaccines, such as the science behind the vaccines (immunity and mutation), the techniques used, and results of clinical trials. These uncertainties have also provoked people to seek information about the vaccine rollout and administration because these topics are are closely connected to when and how people will be vaccinated.

The results of this study show that the discussions about COVID-19 vaccines are multifaceted, and the public are actively seeking and sharing information about them. It is important for public health agencies to understand the major public interests and concerns regarding vaccination, that is, the major factors that would affect vaccine acceptance and hesitancy. In this way, they could establish appropriate strategies to facilitate public communication. Our study shows that the major spikes in vaccine discussions corresponded to reports of the breakthroughs of vaccine development as they appeared in the news. Public health agencies need to pay attention to this pattern and monitor the discussions on the web on days when major news is reported about vaccine development or other significant events, such as a report of severe adverse events from a particular vaccine. These agencies could develop immediate responses based on identifying the instant reactions—and the dominant emotions and topics—on social media. In this study, we found that trust is the predominant emotion regarding vaccines, which is promising and reassuring for the public health agencies promoting vaccination. For individual themes and topics, public health agencies should pay close attention to the discussions about vaccine knowledge and administration to close the information gap between the needs of the public and the information the agencies have provided.

### Limitations

By examining how topics and sentiments evolve along the timeline of the pandemic and vaccine development, one can see the correspondence of the volume of tweets and major research breakthroughs in the news. Although it is clear that the number of tweets shows a major spike at the news of Pfizer reporting its high vaccine efficacy in early November, a statistical analysis may provide more details about whether there are significant differences. Although it is not the focus of this study, close examination of Twitter users may provide more meaningful information, such as how the contents shared by different kinds of users vary and whether certain types of users are more likely to post or comment on certain topics. In addition, this study may have geographic bias in the examined tweets, as is observed in many other studies of unstructured textual data [38]. As a result, a given data set could overrepresent some geographic areas. Last but not least, because Twitter users are not representative of the US population [39], our data set could be overly representing a subset of the population with specific characteristics. Therefore, the findings of this study should be generalized with great caution.

### Conclusion

This study identifies the major topics and sentiments about COVID-19 vaccine–related issues discussed on social media. It also examines the changes in these topics and sentiments over time to better understand the larger trend. Among the 16 distinct topics, opinions about vaccination was the most common and remained so over time. As vaccine development progressed around the world, the dominant topics also shifted. Instructions on getting the vaccine became the most discussed topic around early January 2021. The discussion of COVID-19 vaccination on social media was largely driven by major news events about COVID-19 vaccines and mirrored the active new topics in mainstream media. Also, the discussion has a global perspective. The overall sentiment was increasingly positive over time, and trust was the predominant emotion, which shows that social media discussions may imply higher acceptance of COVID-19 vaccines compared with previous vaccines. Due to the timeline of our data set, in this study, we did not further examine the sentiments about specific brands of vaccines. We would expect the discussions to be different when using different brand names to search for tweets and conduct sentiment analysis. Particularly, after administration of the Johnson & Johnson vaccine was paused by the CDC, there could be a surge of related discussions, and a topic about the side effects of the vaccines could emerge. Therefore, further study in this line is highly recommended.

## Appendix

Multimedia Appendix 1 Coherence scores of the different numbers of topics.

## Abbreviations

ASCII: American Standard Code for Information Interchange
CDC: US Centers for Disease Control and Prevention
DTM: document-term matrix
FDA: US Food and Drug Administration
JSON: JavaScript Object Notation
LDA: latent Dirichlet allocation
