# Supplementary figures and images for: COVID-19 Vaccine–Related Discussion on Twitter: Topic Modeling and Sentiment Analysis
Source: J Med Internet Res. 2021 Jun 29;23(6):e24435. doi: 10.2196/24435 (PMC8244724; doi:10.2196/24435)

**Multimedia Appendix 1. Coherence scores of the different numbers of topics.**


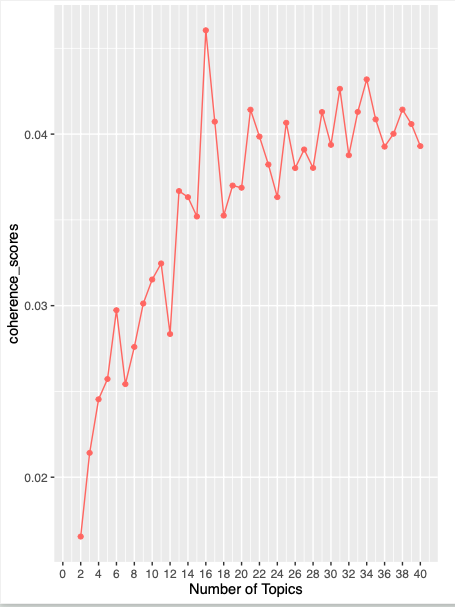

Supplement: Multimedia Appendix 1 [file jmir_v23i6e24435_app1.docx]
